# Silicene synthesis beyond graphene: a critical review of methods, stability challenges, and scalable pathways

**DOI:** 10.1039/d6na00372a

**Published:** 2026-07-29

**Authors:** Md. Mohi Uddin, Mohammad Asaduzzaman Chowdhury, Mohammad Rashed Mia, Md. Siam Ali Shikder

**Affiliations:** a Department of Mechanical Engineering, Dhaka University of Engineering and Technology (DUET) Gazipur 1700 Bangladesh Engr.Md.MohiUddin@Outlook.com

## Abstract

Silicene, a buckled honeycomb-lattice allotrope of two-dimensional (2D) silicon, has attracted strong interest as a candidate for post-silicon electronics, owing largely to its compatibility with existing silicon-based fabrication methods and its theoretically predicted massless Dirac fermions. However, the material suffers from three persistent obstacles that prevent its device-level deployment. First, a naturally layered silicon parent crystal does not exist, which makes top-down exfoliation fundamentally difficult. Second, silicene is rapidly oxidised in air and moisture, which limits its ambient handling. Third, no current synthesis route has been able to deliver scalable production, defect-controlled layers, and preservation of the intrinsic electronic structure simultaneously. The main objective of this review is to compare, within a unified framework, the four widely investigated silicene synthesis routes: electrochemical exfoliation, epitaxial growth on metallic substrates, plasma-enhanced chemical vapour deposition (PECVD), and Zintl phase topotactic deintercalation, against three application-driven criteria, namely scalability, ambient stability, and electronic fidelity. The lithiation delithiation electrochemical route is found to produce few-layer nanosheets of approximately 2.4 nm thickness under ambient conditions, yet the dimensional control is poor, and the product degrades almost instantly in air. The epitaxial growth on Ag(111) is found to provide the highest crystallographic precision; however, the Dirac cone is suppressed by the strong silicene Ag hybridisation, whereas the Au(111) substrate is shown by simulation to preserve the cone with reduced buckling. The PECVD route is found to yield hydrogenated silicene with measurable oxidation resistance, as confirmed by X-ray photoelectron spectroscopy (XPS), but the method demands tight control over multiple deposition parameters and requires a mandatory Al_2_O_3_ capping layer. The Zintl phase route is found to produce multilayer silicene flakes up to 100 µm in lateral extent, and the recent vacuum–nitrogen-assisted synthesis (VANS) variant dramatically reduces the reaction time from five days to the order of minutes, while operating at room temperature and preserving the structural properties of the conventional product. From the above comparison, it is concluded that no single method satisfies all three criteria concurrently. A hybrid pathway, in which VANS grown silicene is encapsulated by PECVD-derived Al_2_O_3_ capping, is therefore proposed as a promising near-term route toward scalable and air-stable silicene.

## Abstract 2

No silicene synthesis route yet reconciles scalability, air stability and electronic fidelity. Combining VANS growth with PECVD-derived Al_2_O_3_ capping is put forward as the most practical near-term route to scalable, air-stable silicene.

## Graphical abstract

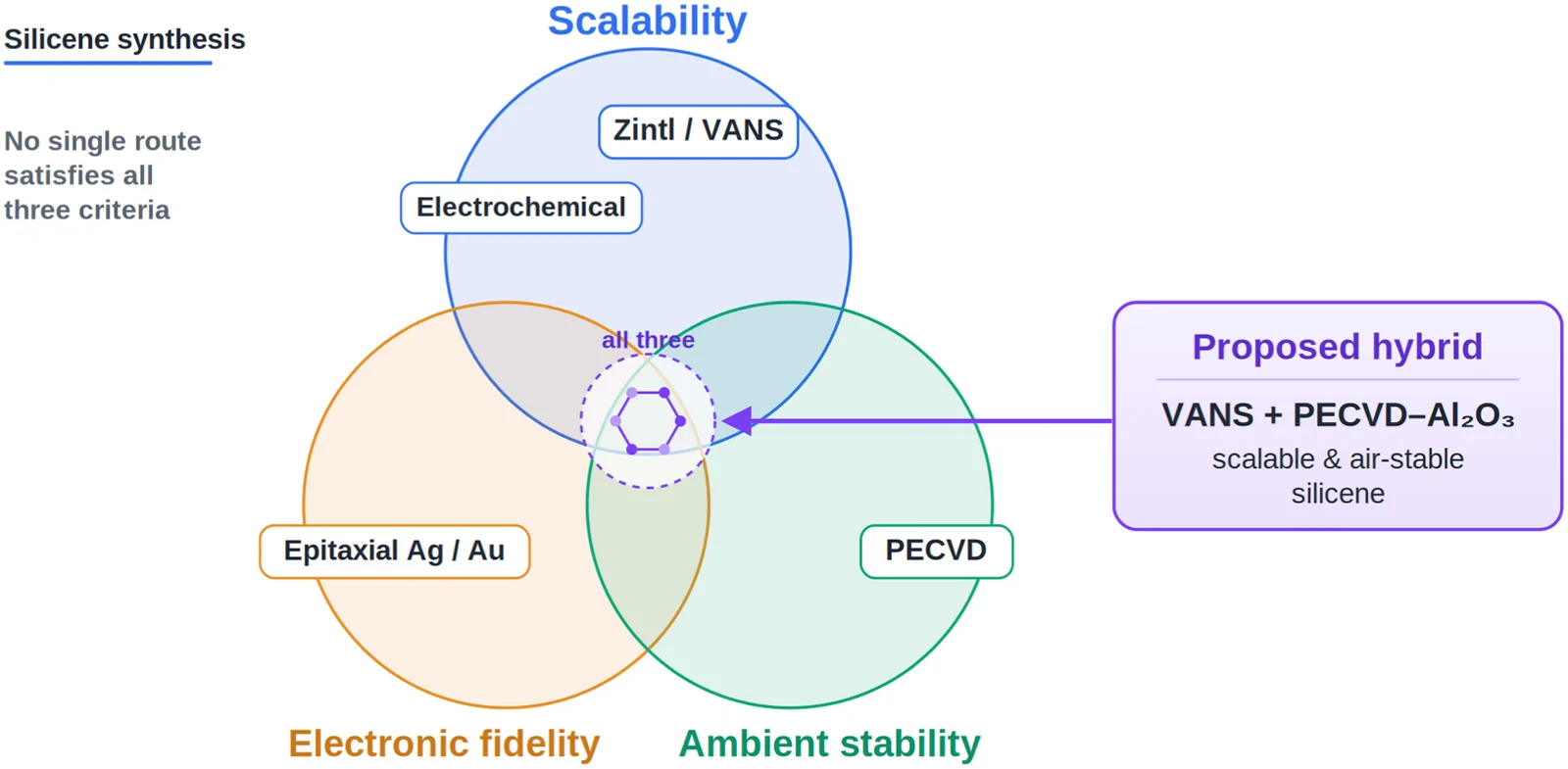

## 1. Introduction

Two-dimensional (2D) materials have become a cornerstone of modern materials research, which is found to have broad application in fields ranging from energy harvesting and protective coatings to automotive engineering, electronics, optoelectronics, and sensing.^1–3^ Since the mechanical exfoliation of graphene in 2004,^4^ the field has grown to encompass a rich family of related 2D allotropes. This group includes hexagonal boron nitride, transition metal dichalcogenides, phosphorene, germanene, and stanene, each having its own unique properties in terms of its electronics, mechanics, and optics.^5–9^ From this group, the material that has received significant attention is silicene, mainly because it can be thought of as an analogy to graphene in terms of silicon.^10–14^

This review highlights experimentally prepared silicene but there exist many other allotropes of silicon in 2D forms, some of which include silicite,^15^ popsilicene,^16^ and irida silicene.^17^ These show interesting predicted electronic properties but remain outside the scope of the present review, as experimental synthesis and validation are still limited.

Unlike graphene, silicene adopts a slightly buckled honeycomb lattice, in which the two silicon sub-lattices are vertically offset by approximately 0.44 Å, as first predicted theoretically by Takeda and Shiraishi.^18^ This buckling is not a structural defect but a consequence of the preference of silicon for sp^3^-like hybridisation, and it has two important electronic consequences. First, a strong spin–orbit coupling is introduced, which is predicted to support an experimentally accessible quantum spin Hall effect.^10,14^ Second, the buckling creates a tunable band gap under an external electric field, which is a feature that is not available in planar graphene.^11,14^ Therefore, silicene is regarded as one of the most attractive candidates for next-generation spintronic and low power electronic devices.^13,14^

Despite the theoretical attractiveness described above, silicene faces three fundamental obstacles to practical deployment. First, unlike graphite, silicon does not possess a naturally layered bulk counterpart, and therefore, top-down exfoliation is inherently difficult.^19^ Second, silicene is highly reactive under ambient conditions and is oxidised rapidly upon exposure to air or moisture.^13^ Third, no existing synthesis method achieves bulk production, defect-free layers, and preservation of the intrinsic electronic properties simultaneously.^10,19^ These three limitations form the central motivation of the present review.

To address these limitations, four synthesis routes have been developed and refined over the past decade. These are: (i) epitaxial growth on a metallic substrate,^20,21^ (ii) electrochemical exfoliation of lithiated silicon,^22^ (iii) topotactic deintercalation of Zintl phase precursors,^23^ and (iv) plasma-enhanced chemical vapour deposition (PECVD).^24,25^ Each route addresses one or two of the obstacles described above, but none of them addresses all three, and a systematic comparison is therefore necessary to guide the rational design of future silicene synthesis strategies.

Several reviews on silicene synthesis and properties have been published in recent years: Masson and Prévot^19^ surveyed the epitaxial growth and the substrate-induced reconstructions; Shan *et al.*^10^ summarised the electronic structure and the device outlook; Ghosal *et al.*^12^ reviewed the transport characteristics for bio-sensing; Molle *et al.*^13^ reviewed silicene derivatives and their transistor integration; and Reslan *et al.*,^26^ together with Galashev^27^ surveyed silicene as an anode material for lithium-ion batteries. A side-by-side evaluation of the four widely investigated silicene synthesis routes against a unified set of performance criteria has not yet been reported, to the knowledge of the authors. While existing reviews have focused on specific aspects, the present review offers a broader perspective with a threefold contribution: (i) a unified comparative framework (Table 1) for all four major routes; (ii) consistent evaluation against three criteria scalability, ambient stability, and electronic fidelity; and (iii) explicit engagement with the first-principles energetics study by Pan and Chou,^28^ rather than a cursory acknowledgement.

**Table 1:** A side-by-side comparison of the four silicene synthesis routes across seven application-driven attributes. This table defines the unified framework for the paper. Primary sources are cited in the column headers, and all attribute values are sourced from these references unless specified otherwise in the text

Attribute	Electrochemical Exfoliation^22,29^	Epitaxial growth (Ag/Au)^28,30–32^	PECVD^24,25^	Zintl/VANS^23,33^
Scalability	Moderate	Low	Moderate–High	High
Air stability	Very poor	Poor	Good (with Al_2_O_3_ cap)	Moderate
Electronic fidelity	Disordered	Ag: suppressed; Au: preserved	Hydrogenated	Multilayer, H-terminated
Lateral size	30–100 nm	Sub-monolayer domains	Wafer scale	2–100 µm
Thickness	2.4 nm	Monolayer	Mono/multilayer	<10 µm
Synthesis time	Hours	Hours (UHV)	10–20 min	5 d (conv.)/minutes (VANS)
Capital cost	Low	Very high	Moderate	Low/moderate

Table 1 reveals three key observations that guide the method-specific discussions below. First, the scalability axis splits the four routes almost evenly: epitaxial growth falls into the low-scale regime, Zintl/VANS into the high-scale regime, and electrochemical and PECVD fall somewhere in between. Second, no single route ranks highest across all three primary criteria scalability, air stability, and electronic fidelity pointing to a fundamental trade-off. Third, we include capital cost a metric rarely seen in synthesis reviews because it strongly affects each route's industrial viability. With this framework in hand, we now turn to the detailed discussion of each method. The four synthesis methods are discussed in detail in Sections 2.1-2.4, with explicit reference to Table 1 in each subsection. The main trade-offs among the methods are analysed in Section 3. Finally, the conclusions and the recommendations for future research are summarised in Section 4.

## 2. Synthesis methods

### 2.1. Electrochemical exfoliation

It can be seen in Table 1 that the electrochemical exfoliation route is low in cost, moderate in scalability, and carries out the reaction under ambient conditions. However, the air stability of the product is rated as very poor, and the dimensional control is limited. The method is based on the lithiation/delithiation chemistry of silicon nanopowder, as described by Zhang *et al.*.^22^ The authors use the conservative terminology “silicene-like” for their product, in acknowledgement of the possible presence of residual hydrogen or amorphous regions in the as synthesised sheets.

**Fig. 1:**
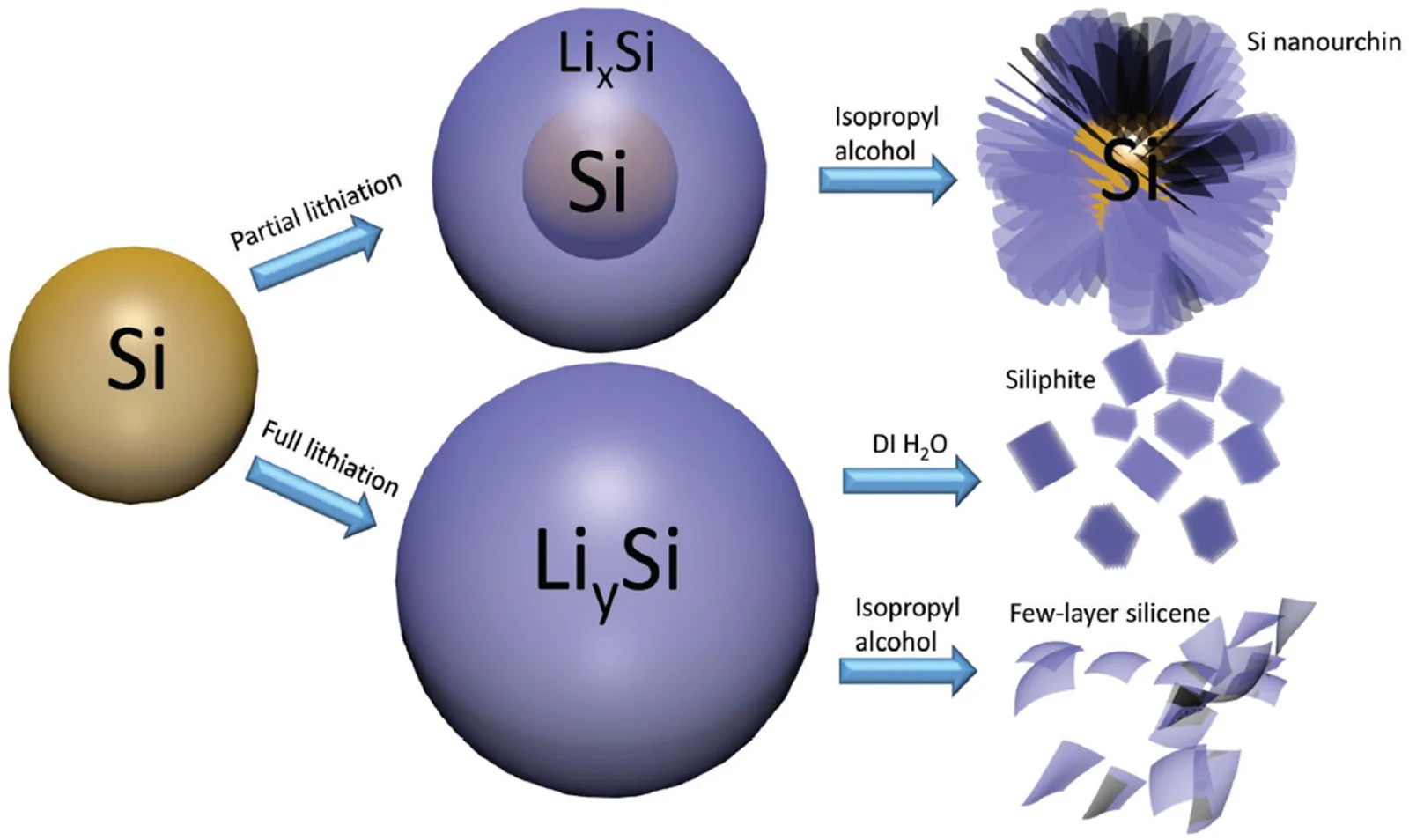
This schematic illustrates how lithiation and delithiation of silicon nanopowder work. The left branch shows lithiation, where Si nanopowder transforms into the Li_*x*_Si alloy. The right branches depict the three products that form during delithiation: (i) silicon nanourchin and (ii) siliphite, which appear when deionised water drives fast kinetics; and (iii) few-layer silicene, which emerges when isopropyl alcohol slows things down enough to preserve the 2D sheet structure.^22^

In this method, silicon nanopowder is used as the cathode and as the silicon source. The nanopowder is produced mechanically by dry ball milling. The lithiation step is performed by discharging a Li-ion coin cell, which produces a lithium silicon alloy of nominal composition Li_*x*_Si. The silicene is then extracted from the Li_*x*_Si alloy by rinsing the electrode in an ultrasonic bath using isopropyl alcohol as the rinsing medium. The choice of rinsing medium governs the morphology of the final product. When deionised water is used in place of isopropyl alcohol, the delithiation reaction proceeds too quickly, yielding silicon nanourchins and siliphite instead of few-layer silicene.^22^ This kinetic selectivity is illustrated schematically in Fig. 1.

As Fig. 1 shows, the slower kinetics of the isopropyl alcohol rinse is what makes 2D silicene formation possible without this kinetic selectivity, the electrochemical route simply wouldn't work. Moving from mechanism to morphology, the synthesized particles retain an intact silicon core that remains unchanged during the initial reaction. This core is wrapped in a shell of delithiated nanosheets radiating outward, giving the particles their characteristic radial shape hence the name silicon nanourchins.

**Fig. 2:**
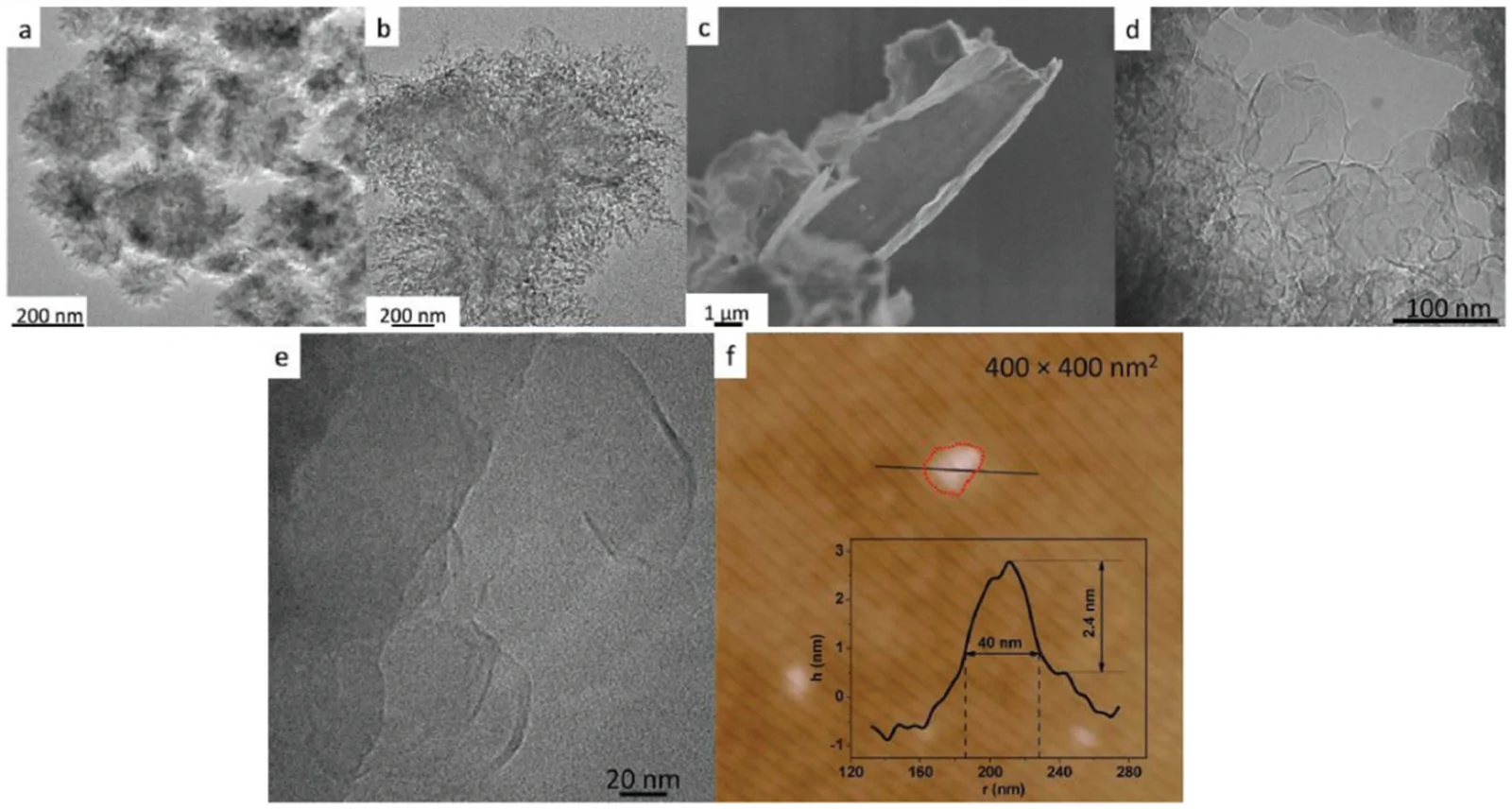
Electron and atomic force microscopy images capture the three delithiation products in detail. (a and b) TEM shows silicon nanourchin, with Si nanosheets radiating from a residual core. (c) SEM reveals siliphite as layered stacks about 10–20 nm thick. (d and e) TEM and HRTEM of few-layer silicene show a wrinkled monolayer structure, roughly 2–3 nm thick and 30–100 nm across. (f) AFM of the same sheet gives a representative thickness of 2.4 nm.^22^

Fig. 2 shows three main things. First, the nanourchin morphology in (a,b) tells us the reaction moves from the surface inwards, with the silicon core acting as a template for the nanosheet shell around it. Second, the graphite-like layered structure of siliphite in (c) points to intermediate structures forming because of kinetic instability. Third and most important the images in (d–f) give three separate pieces of evidence for 2D silicene: the sheet-like shape in TEM, the missing 3D Moiré pattern in HRTEM, and the sub-3 nm thickness measured by AFM.

From the above discussion, it is concluded that the electrochemical exfoliation of lithiated silicon yields few-layer silicene nanosheets with a characteristic thickness of 2.4 nm and lateral dimensions of 30–100 nm. Three significant drawbacks must be noted: (i) high sensitivity to the delithiation medium, (ii) poor control over thickness and yield, and (iii) critical instability in air and moisture requiring inert atmosphere or vacuum storage. Having established the electrochemical baseline, the next section turns to the epitaxial route, in which dimensional control is improved at the cost of scalability.

### 2.2. Epitaxial growth

The epitaxial route delivers the highest crystallographic quality of the four methods, but at the cost of low scalability and high capital expenditure (Table 1). Silicene is grown by direct deposition of Si onto a metallic substrate under ultra-high vacuum (UHV).^19,32^ The substrate choice governs both the structural order and the fate of the Dirac cone. Substrates explored so far include Ag(111),^20,34^ Au(111), Au(110), Ir(111), Pb(111), ZrB_2_, MoS_2_, Ru,^35^ Al(111),^36^ and graphite.^19^ Also, recent advances have demonstrated the formation of Xene heterostructure beyond the growth of isolated silicene layers. Dhungana *et al.* report the epitaxial growth of silicene stanene heterostructure on Ag(111), suggesting that a Xene layer can act as a template for the growth of another concurrently preserving the hexagonal arrangement of the constituent layers. This broadens the concept of epitaxial silicene growth toward engineered multilayer Xene heterostructure for future electronics and heterojunction-based device applications.^37^ This single-phase heterostructure route also begins to address the multiple-phase and domain-size limitations of conventional Ag(111) epitaxy. Of these, Ag(111) has received the greatest attention and is discussed first.

#### 2.2.1. Ag substrate

Before examining the growth mechanism, we should recognize a key complication: silicene on Ag(111) does not form a single, well-defined phase. Depending on the substrate temperature and silicon coverage, at least three distinct superstructures are commonly observed. The (4 × 4) phase, relative to the underlying Ag(111) lattice, is the most studied and widely accepted as the primary silicene overlayer. Then there's the (√13×√13) R13.9° phase, which appears at slightly higher temperatures and brings a different in-plane lattice constant and buckling configuration. Finally, the (2√3 × 2√3) R30° phase emerges at higher coverages though whether this represents true multilayer silicene or a reconstructed alloy surface remains an open question. Each phase has its own characteristic STM height, Raman signature, and critically its own degree of hybridisation with the Ag substrate. This means future studies probing the electronic structure of silicene on Ag(111) must clearly specify which phase is under investigation; otherwise, transport and ARPES data from mixed-phase surfaces become nearly impossible to interpret unambiguously. For the remainder of this section, “silicene on Ag(111)" refers specifically to the (4 × 4) phase unless otherwise stated.^19^

The epitaxial growth of silicene on Ag(111) was first demonstrated by Lalmi *et al.*^34^ in 2010 using atomic-resolved scanning tunnelling microscopy (STM), who reported a highly ordered honeycomb lattice with two sub-lattices offset by ≈ 0.2 Å. Subsequent compelling evidence from combined STM, angle-resolved photoemission spectroscopy (ARPES), and density functional theory was provided by Vogt *et al.*^21^ in 2012. De Padova *et al.* first reported silicene-like growth on Ag(110),^19^ and Ag(111) was subsequently proposed by Gao and Zhao^38^ as providing a better lattice match and a more ordered phase.

**Fig. 3:**
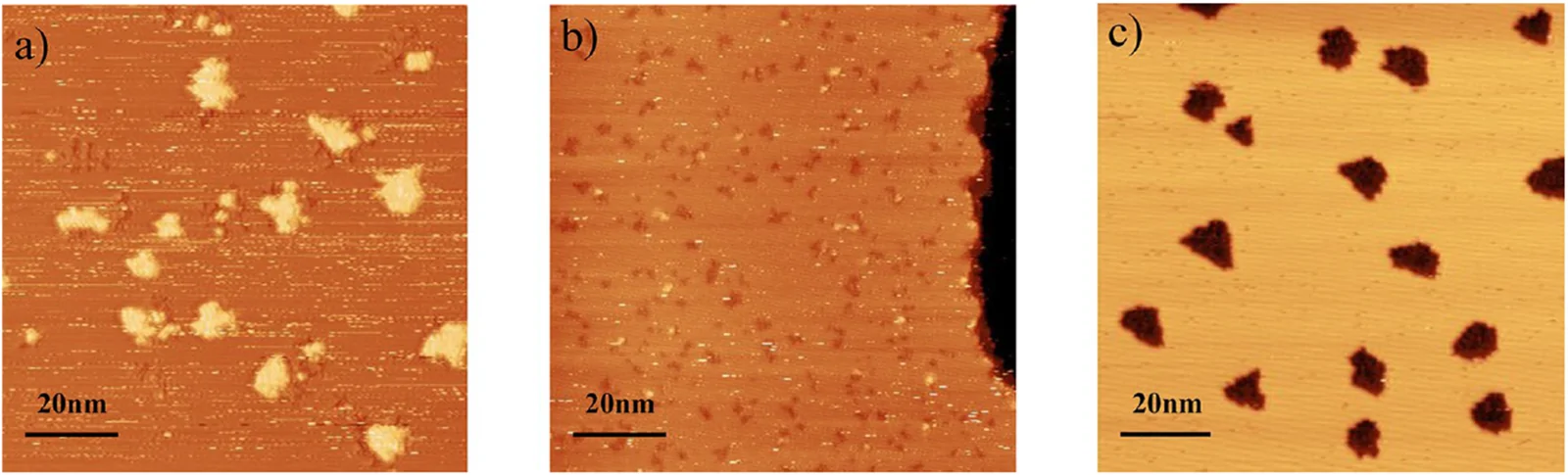
STM images of silicene deposition on Ag(111) at three substrate temperatures. At 198 K (left), Si atoms are inserted into the Ag surface as isolated adatoms. At 300 K (centre), Si islands nucleate and coalesce. At 440 K (right), a continuous honeycomb silicene layer forms, with residual Si island regions. The sequence shows the gradual transition from substrate-inserted to continuous 2D phase across 198–440 K.^40^

The silicene deposition on Ag(111) exhibits two distinct modes, depending on the substrate temperature.^39,40^ At temperatures below 200 K, the deposited silicon atoms are inserted into the Ag substrate itself. A two-dimensional silicene island is then formed first, and further deposition increases the coverage in a manner analogous to homoepitaxial sub monolayer growth.^40^ This low-temperature growth mode is illustrated by the scanning tunnelling microscopy (STM) images in Fig. 3.

**Fig. 4:**
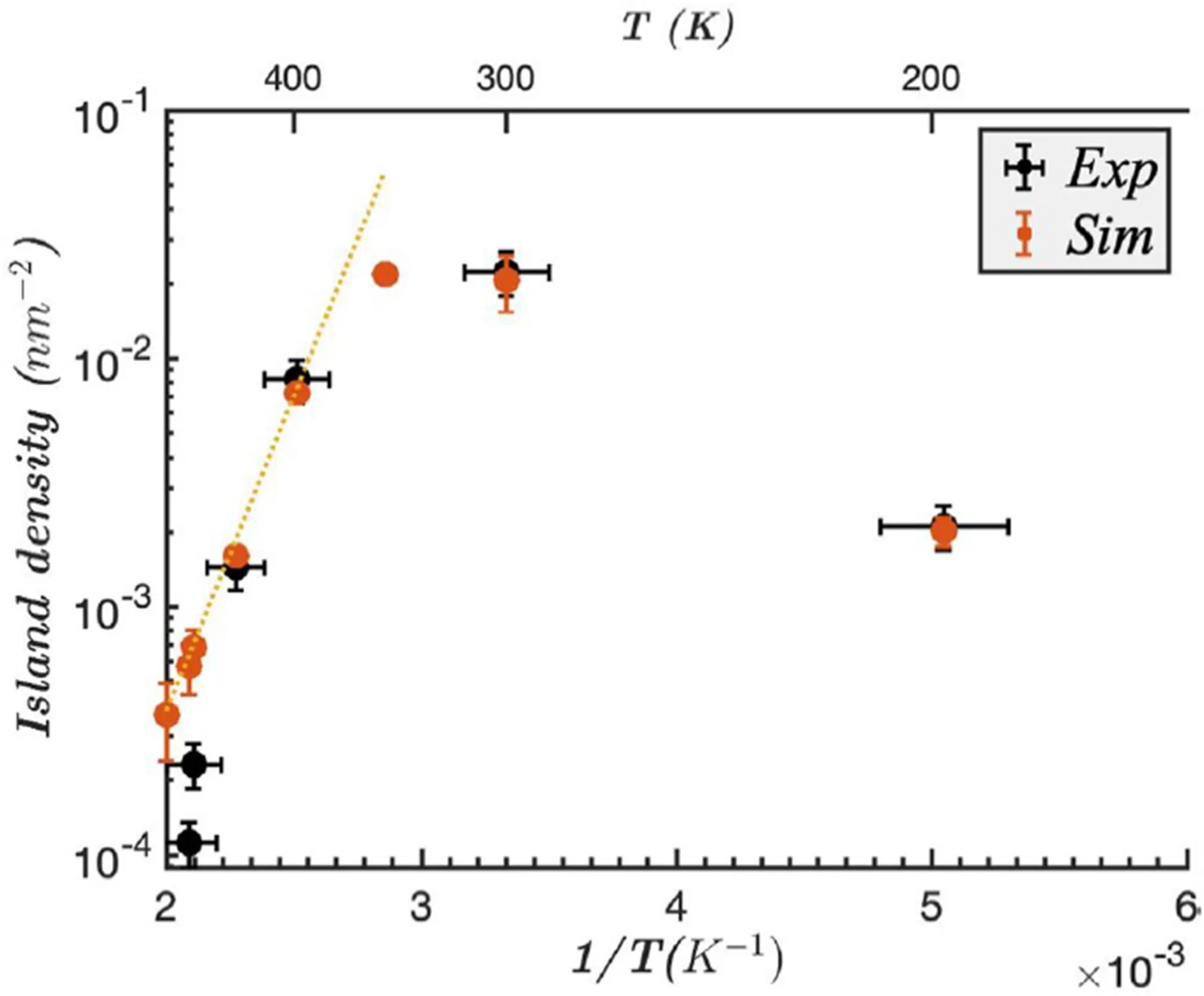
Experimental and simulated silicon island density on the Ag(111) substrate as a function of temperature. The island density is observed to decrease rapidly in the 300–400 K range, as the individual islands coalesce to form a continuous silicene layer. Above 440 K, the island density reaches a low plateau, which corresponds to the fully developed monolayer regime. The agreement between the experimental data points^40^ and the simulation curve obtained by kinetic Monte Carlo modelling^39^ validates the adatom-mediated growth model of silicene on Ag(111).

Fig. 3 demonstrates that the transition from isolated substrate-inserted silicon atoms at 198 K to a continuous silicene monolayer at 440 K is controlled directly by the deposition temperature. In practice, this dependence on temperature is a good thing. It means the quality of silicene can be tuned just by changing the growth temperature. The high temperature regime, though, operates through a completely different mechanism. Once the temperature climbs above 300 K, Ag atoms are driven out of the substrate and head for the step edges, leaving behind vacancies that silicon atoms promptly fill. The steep drop in island density between 300 and 400 K identifies this window as the optimal temperature range for the production of a continuous silicene monolayer. Annealing above 630 K causes progressive dewetting of the silicene layer.^19^ The quantitative temperature dependence of the island density is shown in Fig. 4.

Most importantly, the silicene grown on Ag does not preserve the Dirac cone, because of the strong hybridisation between the silicene and the Ag substrate.^31^ This electronic penalty represents the single largest limitation of the Ag(111) route and motivates the search for alternative substrates.

#### 2.2.2. Au(110) and Au(111) substrates

Jaroch and Zdyb^41^ recently explored the temperature-dependent growth of silicene on ultrathin Au films, using LEEM and LEED to characterize the process.^41^

**Fig. 5:**
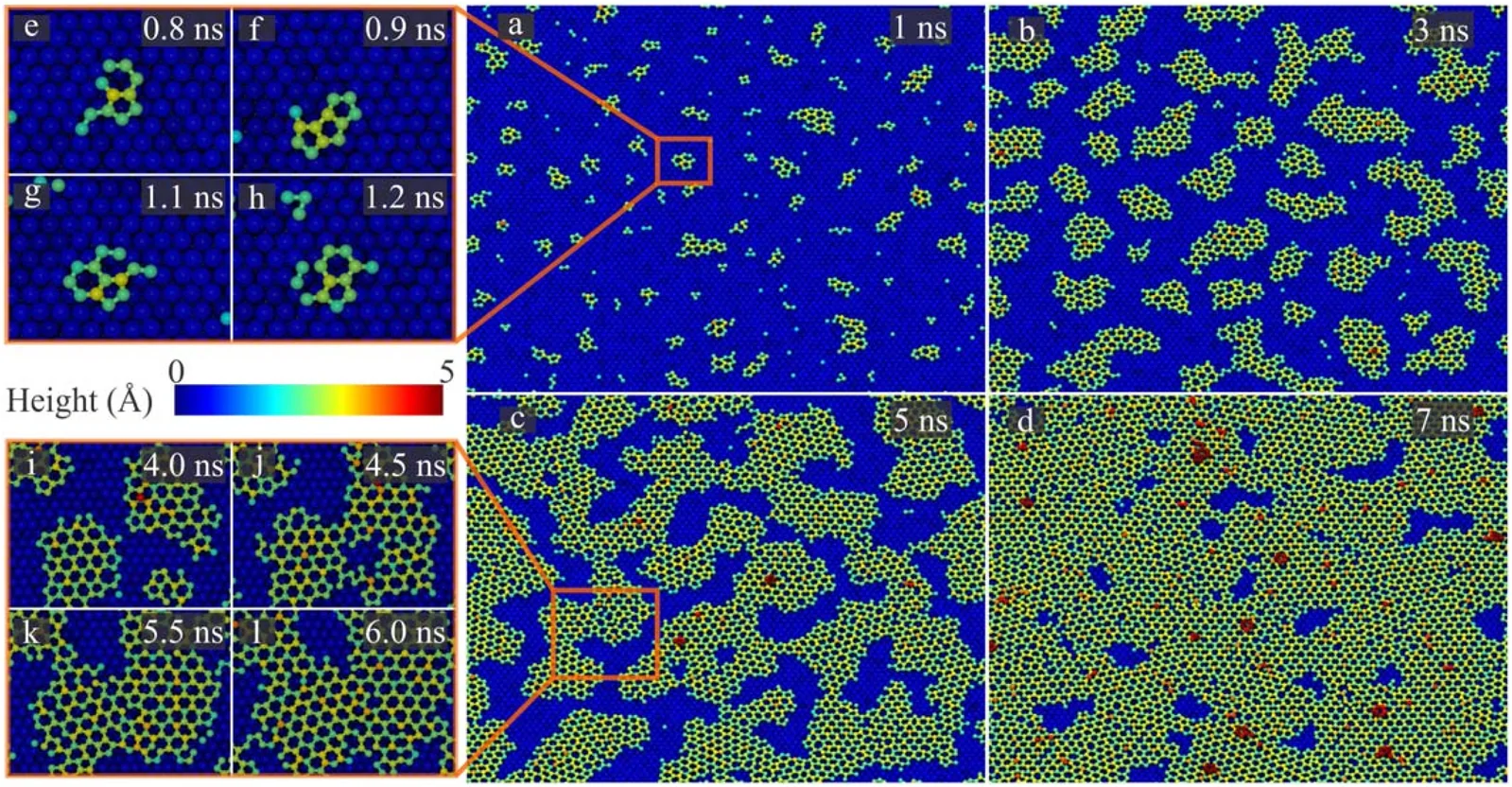
Molecular dynamics snapshots of silicene island growth on Au(111) and Au(110) as a function of time. (a and b) Initial nucleation of small silicene islands. (c and d) Coalescence into larger domains within nanoseconds. (e–h) Magnified views of isolated islands with atomic-scale rim structure. (i–l) High temporal resolution snapshots of island coalescence. Red zones indicate regions of greater silicene height (local multilayer stacks).^30^

Building on these experimental foundations, Barboza *et al.*^30^ recently addressed the Dirac cone shortcoming through molecular dynamics simulations on the Au(111) and Au(110) substrates, and it was shown that the Dirac cone is preserved far more effectively on gold. The time evolution of the silicene island growth on the two Au substrates is displayed in Fig. 5.

**Fig. 6:**
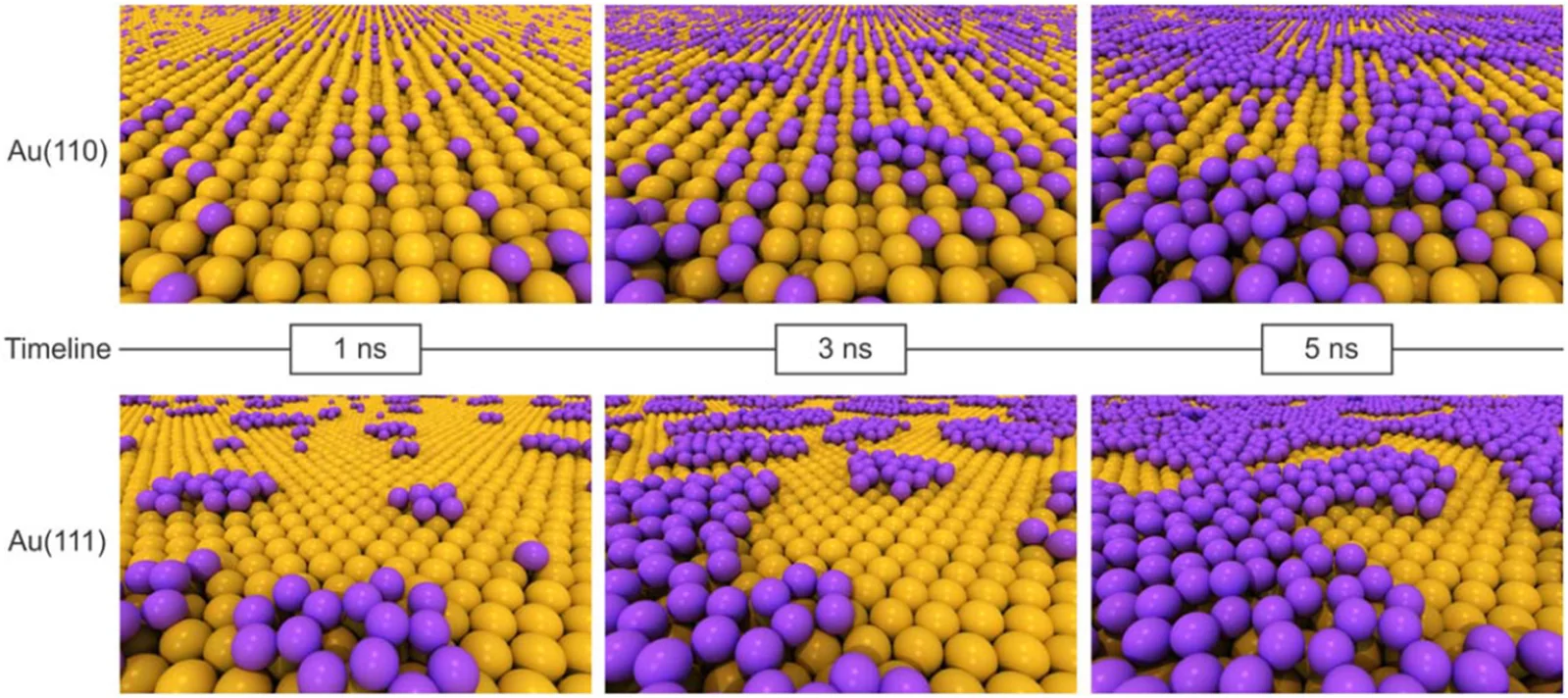
MD snapshots of silicene deposition on Au(110) and Au(111). Violet atoms: silicon; yellow atoms: gold. On Au(110), a single atomic diameter Si wire forms first along the surface ridges, and subsequent adatoms build up over 3 ns into an elongated strip-like island. On Au (111), Si deposition is uniform and isotropic from the outset, a direct consequence of the smooth fcc (111) close-packed termination.^30^

Fig. 5 reveals that the qualitative growth sequence on the two Au surfaces is similar: initial nucleation (Fig. 5a and b), growth of isolated islands (Fig. 5e–h), and coalescence into a continuous 2D layer (Fig. 5c, d and i–ld). The quantitative coalescence kinetics, however, differ between the two substrates. It is found that both growth and coalescence are faster on Au(111) than on Au(110). In both cases, the number of islands decreases with the increase of the temperature, because the smaller islands merge into a single larger island. A more direct visual comparison of the deposition morphology on the two substrates is shown in Fig. 6.

The simulations by Barboza *et al.*^30^ show clearly that the silicene deposited on Au(110) adopts a strip-like morphology, which is in agreement with an earlier experimental observation. This morphology is attributed to the rough atomic-scale corrugation of the Au(110) surface: a single atomic diameter wire is first deposited between the surface ridges, and silicene adatoms are subsequently deposited over a further 3 ns to form an elongated island. In contrast, the Au(111) surface is smooth and close-packed face-centred cubic, and a uniform silicene layer is produced from the outset. The quantitative impact of the surface topography on the silicene bond length is summarised in Table 2.

**Table 2:** The silicene bond length on Au(110) and Au(111) substrates at three deposition temperatures, reported as Au(110)/Au(111) pairs (Å). The data are drawn from the molecular dynamics study of Barboza *et al.*^30^

Deposition rate (atoms ns^−1^)	300 K Au(110)/Au(111)	500 K Au(110)/Au(111)	700 K Au(110)/Au(111)
1000	2.444/2.414	2.445/2.419	2.409/2.431
2000	2.443/2.422	2.437/2.415	2.432/2.415
5000	2.435/2.420	2.431/2.416	2.425/2.416
10 000	2.434/2.412	2.430/2.411	2.427/2.414

Table 2 reveals two systematic trends. First, across all deposition rates and temperatures, the silicene bond length on Au (110) consistently exceeds that on Au (111) by about 0.01–0.03 Å a clear structural fingerprint of the substrate's corrugation. Second, the bond length is weakly dependent on the deposition rate above 2000 atoms ns^−1^, which indicates that the silicene structure on Au is kinetically robust once a critical deposition rate has been exceeded. In parallel with the bond length analysis, the buckling heights were quantified by Barboza *et al.*.^30^ in the same study. It was found that the buckling is weaker on Au(111) than on Au(110). The buckling heights at three deposition temperatures are reported in Table 3.

**Table 3:** The buckling height of silicene on Au (110) and Au (111) substrates at three deposition temperatures, reported as Au (110), Au (111) pairs (Å). The data are drawn from the molecular dynamics study of Barboza *et al.*.^30^

Deposition rate (atoms ns^−1^)	300 K Au(110)/Au(111)	500 K Au(110)/Au(111)	700 K Au(110)/Au(111)
1000	0.98/0.36	0.88/0.41	0.81/0.47
2000	0.99/0.37	0.85/0.42	0.80/0.48
5000	1.00/0.37	0.86/0.40	0.80/0.45
10 000	1.02/0.37	0.87/0.41	0.83/0.45

From the above discussion, it is concluded that the Au(111) substrate is more promising than Au(110) for silicene growth, because the Dirac cone is preserved, the buckling is closer to the freestanding reference value (0.36–0.48 Å *versus* 0.44 Å freestanding), and a uniform silicene layer is produced. However, it must be emphasised that the molecular dynamics results have not yet been confirmed by direct experiment.^30^

It is further noted that a recent first principles energetics study by Pan and Chou^28^ has argued that silicene, strictly defined as a buckled honeycomb layer preserving the freestanding band structure, cannot be grown on Ag(111): beyond one monolayer, growth is predicted to switch to a three-dimensional Volmer–Weber mode, while the monolayer phase itself does not reproduce the buckling pattern of freestanding silicene. This theoretical claim represents a minority view within a literature in which the majority position is that some form of Si honeycomb layer is indeed produced on Ag(111).^20,21,34,38,42,43^ The controversy remains unsettled, and settling it will require a dedicated experimental re-examination of the silicene/Ag(111) system ideally combining STM, ARPES, and *in situ* Raman measurements.

### 2.3. Plasma-enhanced chemical vapour deposition (PECVD)

Returning to Table 1, PECVD clearly stands out on the stability axis. The silicene it yields is hydrogenated and resistant to oxidation. The method runs at moderate temperatures and avoids the ultra-high vacuum needed for classical epitaxy. The downside, however, is that it's parametrically demanding multiple variables must be carefully optimised.

Beyond the deposition parameters, it is worth being clear about what structural evidence actually exists for the PECVD product. In the original study, the honeycomb structure of the deposited layer was assumed from three observations: (i) Raman spectroscopy showed a G-peak at 516 cm^−1^, which matches a multi-phase silicene monolayer and sits apart from both bulk amorphous silicon (broad peak near 480 cm^−1^) and crystalline Si (520 cm^−1^); (ii) XPS showed no Si^4+^ peak at 102.3 eV (the Si^4+^ binding energy given in ref. 24; Molle *et al.*^13^ put this oxide signature at 103 eV), so the surface had not oxidised; and (iii) Raman mapping at four spots confirmed the film was spatially uniform.^24^ Still, the original work included no atomic-level STM imaging and no diffraction data from LEED or XRD. So the honeycomb assignment rests on indirect evidence Raman phonon modes rather than real-space imaging of atomic positions. In other words, while the Raman data point to a silicene-like phase, they cannot rule out a hydrogenated amorphous or nano-crystalline Si film instead.

In the PECVD method reported by Jugdersuren *et al.*,^24^ Ag films of approximately 16 nm thickness are first grown on a fused silica substrate at a base pressure of 2 × 10^−8^ Torr. After cleaning and a deposition temperature of 210 °C, 250 °C, or 290 °C, silicene is deposited using a mixture of H_2_ and SiH_4_. The deposited silicene is capped with Al_2_O_3_ and stored in a desiccator under continuous N_2_ flow.^24,25^

**Fig. 7:**
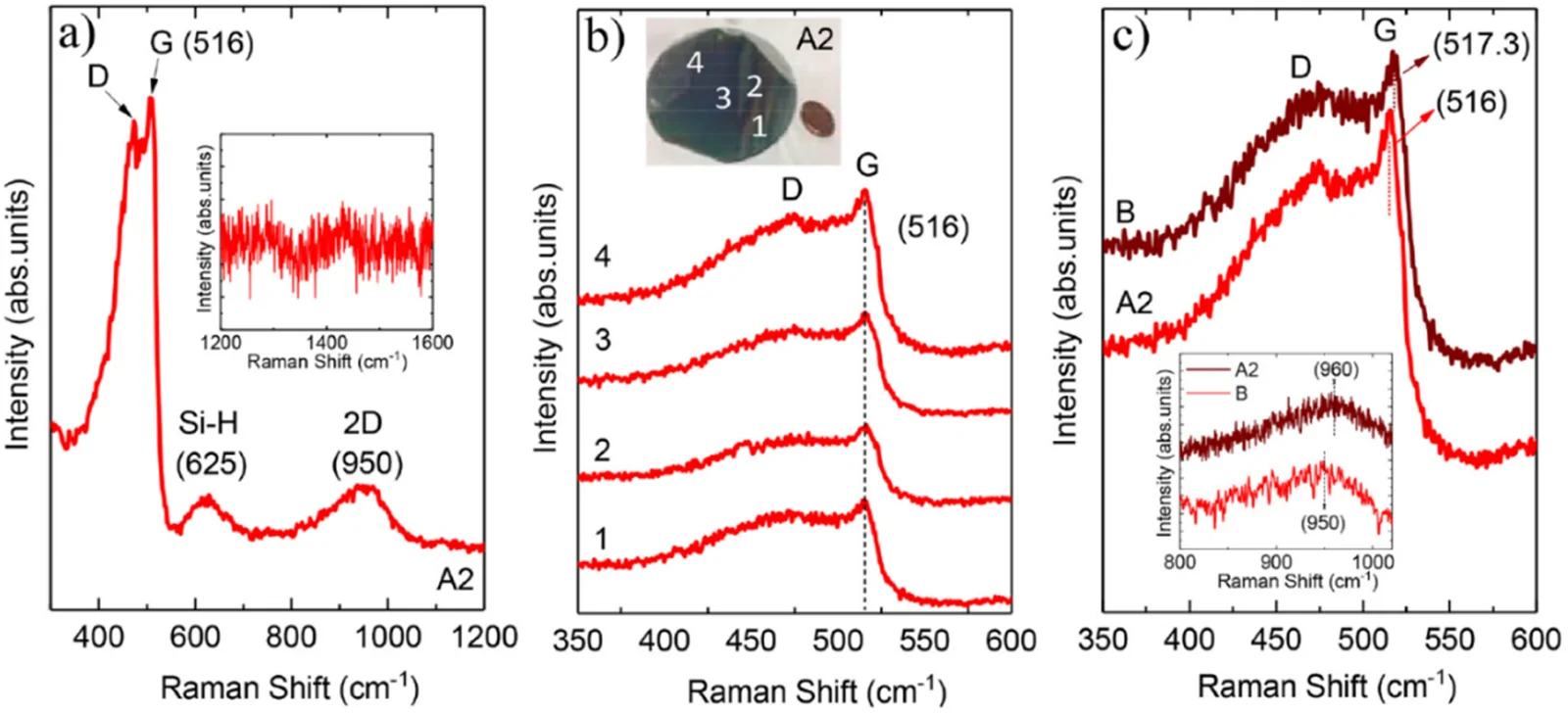
Raman spectra of monolayer PECVD silicene at 250 °C. (a) Full spectrum of sample A2: silicene G-like peak at 516 cm^−1^, Si–H wagging at 625 cm^−1^, second-order defect peak at 950 cm^−1^, and absence of a carbon peak at 1560 cm^−1^. (b) Four spot spectra of A2 confirming spatial uniformity of the G-like, Si–H, and 2D features. (c) Spectrum of sample B (20 min deposition) showing only minor variation attributable to additional multilayer deposition.^24^

**Fig. 8:**
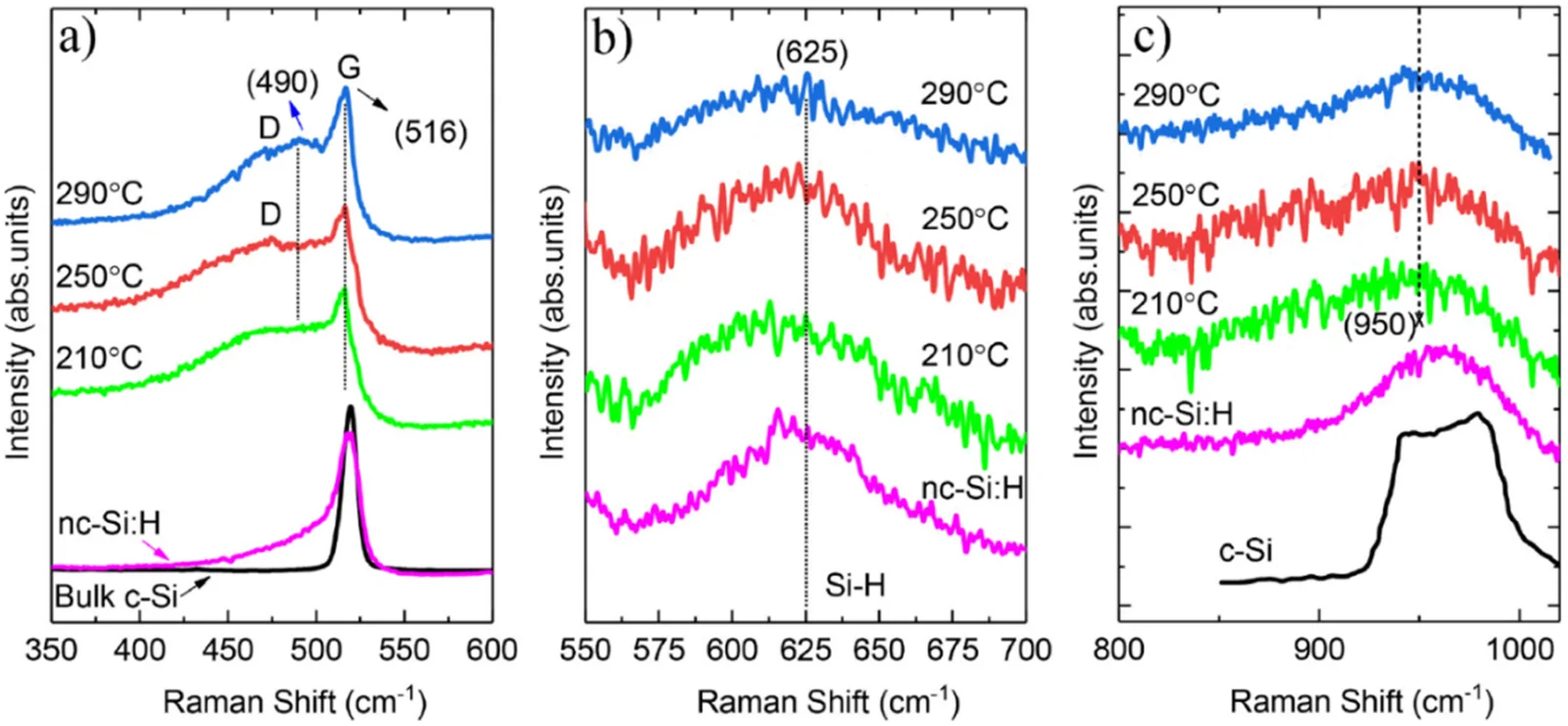
Raman spectra of three PECVD silicene samples: A3 (290 °C), A2 (250 °C), and A1 (210 °C).(a) G-like peak invariant at 516 cm^−1^ across all three temperatures, but growing in intensity with T.(b) Si H wagging peak essentially unchanged. (c) 2D peak also invariant Pink trace noncrystalline silicon; black trace: bulk single crystal Si(111) ref. 24.

Table 4 shows that the experimental design cleanly isolates two variables. The substrate temperature effect is probed by comparing samples A1, A2, and A3, in which all other deposition parameters are held constant. The deposition time effect is probed by comparing sample A2 (10 min) with sample B (20 min), with the same substrate temperature of 250 °C. The structural and electronic consequences of these variations are revealed by the Raman spectra in Fig. 7 and 8.

**Table 4:** Deposition parameters for the four PECVD silicene samples reported by Jugdersuren *et al.*^24^ Samples A1–A3 were deposited at three different substrate temperatures. Sample B was deposited at 250 °C for twice the time used for sample A2 to investigate the thickness dependence

Sample	Substrate T (°C)	H_2_ (SCCM)	SiH_4_ (SCCM)	Power (W)	Pressure (mTorr)	Time (min)	Ag (nm)	Al_2_O_3_ (nm)
A1	210	200	1	90	700	10	16	16
A2	250	200	1	90	700	10	16	16
A3	290	200	1	90	700	10	16	16
B	250	200	1	90	700	20	16	16

A central result of the PECVD work is that the silicene produced by this route retains its structural integrity for a short period of ambient air exposure even in the absence of a protective cap, as verified in a class 100 clean room by X-ray photoelectron spectroscopy.^24^ In addition, the PECVD environment is low cost and chemically inert. The Raman spectra of the PECVD silicene at the optimal temperature of 250 °C are presented in Fig. 7.

Fig. 7 delivers three important pieces of information. First, the G-like peak at 516 cm^−1^ in Fig. 7a is the primary fingerprint of multi-phase monolayer silicene and is consistent with previous reports on molecular beam epitaxy silicene.^44^ Second, the additional peak at 625 cm^−1^ is unambiguously assigned to the wagging mode of the SiH bond, which confirms that the silicene is hydrogenated. This hydrogenation stems directly from the H_2_, SiH_4_ precursor chemistry and is central to the PECVD product's oxidation resistance. Third, the absence of any peak at 1560 cm^−1^ rules out carbon contamination, which would otherwise degrade the silicene's electronic properties. The spatial uniformity seen in Fig. 7b, together with the weak thickness dependence in Fig. 7c, confirms that the PECVD process delivers a homogeneous film across the entire sample. We now examine the temperature dependence of the PECVD silicene through the Raman spectra in Fig. 8.

In Fig. 8, the G-like peak position is invariant at 516 cm^−1^ across all three deposition temperatures, demonstrating that the silicene structure is independent of processing temperature in the range 210–290 °C. The oxidation resistance of the PECVD silicene was verified by XPS: no Si^4+^ peak at 102.3 eV is detected, confirming resistance to oxidation under the measurement conditions.^24,25^ From the above discussion, the PECVD route provides a partial solution to the air stability problem of silicene, at the cost of plasma process complexity and a mandatory Al_2_O_3_ capping layer for long-term storage.

### 2.4. Zintl phase deintercalation

Turning once more to Table 1, it can be seen that the Zintl phase route offers the strongest scalability among the four silicene synthesis methods. In this approach, silicene is produced by the topotactic deintercalation of calcium from the layered Zintl phase precursor CaSi_2_.^23,33^ The method is intrinsically bottom-up from a bulk precursor and is therefore free from the UHV requirement of the epitaxial route.

The commercial CaSi_2_ powder must first be purified from calcium oxide contaminants (CaO, Ca_3_SiO_3_) and calcium silicates (Ca_14_Si_19_), as described by Kozma *et al.*^23^ The purified CaSi_2_ is evacuated for 2 h in a Schlenk line to remove adsorbed gases. A degassed aqueous HCl solution is then added to the CaSi_2_ under vacuum, and the mixture is held at −30 °C under nitrogen for 5 days, during which the topotactic deintercalation of Ca^2+^ proceeds. A yellow-green product is obtained, filtered, washed with dry methanol, and dried under vacuum. The silicene is then stored at −20 °C under nitrogen. This classical procedure is referred to as the conventional Zintl phase route.

**Fig. 9:**
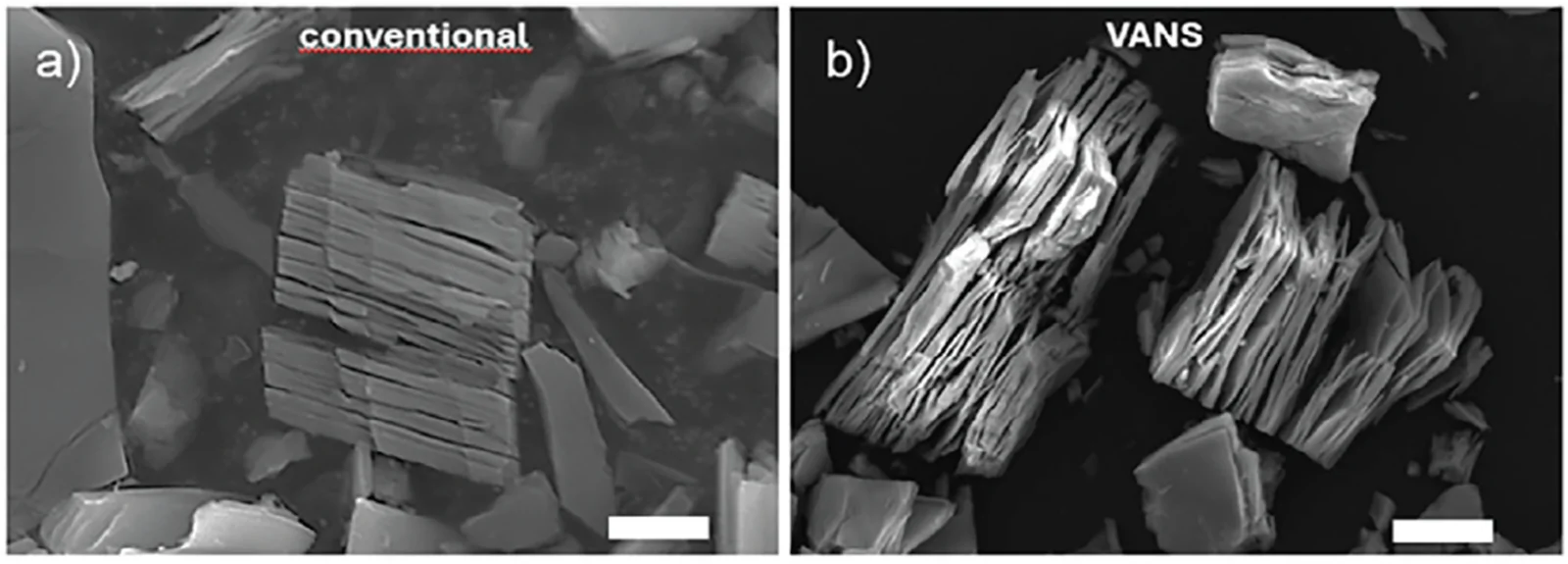
SEM comparison of silicene from (a) the conventional five-day Zintl phase process and (b) the VANS process. Both show multilayer flake morphology with lateral dimensions of 2–3 µm up to 100 µm. The VANS product in (b) is morphologically indistinguishable from (a), despite a reduction of synthesis time from five days to minutes.^23^

A more recent and significantly faster modification, the vacuum nitrogen-assisted synthesis (VANS), was reported by Kozma *et al.*.^23^ The VANS route reduces the reaction time from 5 days to the order of minutes at room temperature. The key process innovation is the repeated cycling of vacuum and nitrogen atmospheres, which efficiently removes the hydrogen generated by the deintercalation reaction. The resulting silicene has been shown by structural characterisation to be equivalent to the conventional product.^23^ A direct SEM comparison of the silicene produced by the two routes is presented in Fig. 9.

**Fig. 10:**
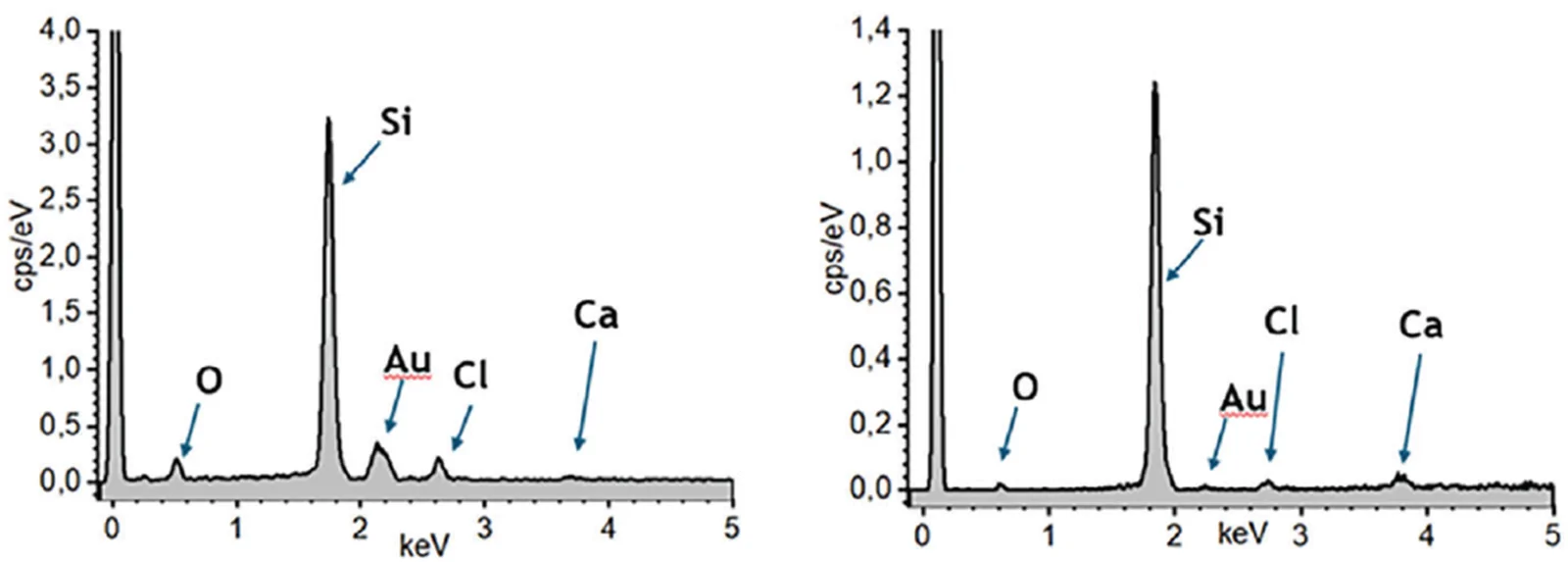
EDS spectra of silicene from (a) the conventional route and (b) the VANS route. The dominant Si Kα peak confirms silicene as the majority phase. The weak Ca Kα peak is attributed to residual Zintl phase precursor not fully deintercalated. The absence of oxygen peaks indicates that oxygen contamination is below EDS detection.^23^

Fig. 9 delivers a striking confirmation that the 600-fold reduction in synthesis time achieved by VANS does not come at the cost of structural quality. Both the conventional and the VANS products exhibit the same multilayer flake morphology, with lateral dimensions ranging from 2–3 µm up to 100 µm, and with thicknesses up to 10 µm depending on the layer count. In both cases, most of the flakes are thinner than 10 µm. This morphological equivalence is the key finding that validates VANS as a practical, scalable route for silicene synthesis. With morphology established, we next compared the elemental purity of the two products. Energy-dispersive X-ray spectroscopy (EDS) was used to quantify the residual calcium content in each.^23^ It was found that only a small Ca signal is detected in both routes. The EDS spectra of the two products are presented in Fig. 10.

Taken together, Fig. 9 and 10 demonstrate that VANS is morphologically and compositionally equivalent to the conventional Zintl phase route, while reducing the synthesis time by approximately 600-fold. This combination of high scalability and chemical purity makes VANS the most practical of the four silicene synthesis routes for large-area production.

## 3. Discussion

Having evaluated each of the four synthesis routes against the comparative framework in Table 1, we now step back from the descriptive summary of Section 2 and draw out the cross-cutting arguments that, in our view, govern the present state of the field. We argue that silicene synthesis is fundamentally limited by a three-way trade-off among structural quality, ambient stability, and scalability.

### 3.1. Quantitative cross-method comparison

A quantitative comparison brings the trade-off into sharp focus. The electrochemical route produces sheets of 30–100 nm lateral extent and ≈2.4 nm thickness,^22^ corresponding to an aspect ratio of only ≈15 : 1, two orders of magnitude lower than the wafer-scale lateral extent achievable by PECVD^24^ and more than three orders of magnitude lower than the 100 µm lateral extent of Zintl phase flakes.^23^ The buckling heights vary by a factor of approximately two across substrates: Au(111) simulations give 0.36–0.48 Å, close to the freestanding reference of 0.44 Å;^18^ Au(110) gives 0.80–1.02 Å;^30^ and Ag(111) introduces the additional penalty of silicene Ag hybridisation that suppresses the Dirac cone entirely.^31^ VANS is the only currently available pathway delivering both industrial-scale throughput and ≥10 µm lateral dimensions, although at the acknowledged cost of electronic fidelity.

### 3.2. Why electrochemical exfoliation continues to underperform

Despite a decade of follow-up studies after Zhang *et al.*,^22^ the electrochemical exfoliation route has not produced silicene with controlled layer number, large lateral extent, or ambient stability. We argue that this is a fundamental kinetic limitation of the lithiation/delithiation chemistry. Because silicon nanopowder is structurally heterogeneous, the delithiation kinetics are inherently non-uniform across an ensemble, resulting in a broad product distribution of nanourchin, siliphite, and silicene-like sheets.^22^ On balance, the electrochemical route is best regarded as a scalable source of few-layer silicon nanosheets for non-electronic applications, for instance, Li-ion battery anodes^26,27^ rather than as a route to device-grade silicene.

### 3.3. The Pan-Chou controversy and the limits of the current evidence

The recent first-principles energetics study by Pan and Chou^28^ concluded that silicene, strictly defined as a buckled honeycomb layer preserving the freestanding band structure, cannot be grown on Ag(111): beyond one monolayer, growth switches energetically to a three-dimensional Volmer Weber mode, and the monolayer phase does not exhibit the buckling pattern of freestanding silicene. This is a minority theoretical view. The majority view, supported by more than a decade of STM, LEED, ARPES, and Raman evidence,^20,21,34,38,42,43^ is that some form of Si honeycomb layer is indeed produced on Ag(111). The direct observation of Dirac-dispersed bands by Chen *et al.*^42^ is the strongest single piece of experimental evidence against the Pan Chou claim. We take the view that the Pan Chou study should be read not as a refutation of silicene on Ag(111), but as a sharp reminder that what has been called “silicene” on Ag(111) may have significant structural differences from the freestanding monolayer.

### 3.4. What VANS has not yet demonstrated

The VANS protocol of Kozma *et al.*^23^ represents, in our assessment, the most significant silicene synthesis advance of the past five years on the scalability axis. However, the structural equivalence of VANS and conventional Zintl phase products is established by SEM, EDS, XRD, and FTIR.^23^ What is not reported is any direct probe of the electronic structure: no ARPES, no transport measurement, no scanning tunnelling spectroscopy. Independent transport measurements on VANS grown silicene, analogous to those of Tao *et al.*^45^ on epitaxial silicene, would either validate or undermine VANS as a route to electronic-grade material. As a primarily growth-focused study, the work of Kozma *et al.* reasonably concentrates on overcoming the key limitations of conventional topotactic deintercalation, so that a detailed electronic characterisation remains an open and worthwhile direction rather than a deficiency. VANS also offers clear practical advantages for gram-to kilogram-scale flake production and for applications that are not accessible to epitaxial silicene.

### 3.5. The hybrid hypothesis: a testable proposal, not a demonstration

We propose, as the main constructive contribution of this review, that a hybrid route combining VANS growth (for scalability) with PECVD-derived Al_2_O_3_ encapsulation (for air stability) is a promising near-term pathway toward scalable, ambient-stable silicene, and one of several stabilisation strategies currently under investigation. We emphasize that this is a hypothesis, not a demonstrated technology. The Tao *et al.*^45^ work is directly cautionary on this point: even Al_2_O_3_ capped silicene was found to degrade readily once the underlying Ag film was removed, and monolayer silicene-based field-effect transistors maintained their ambipolar behaviour only on a minute-timescale. Few- and multi-layer silicene devices, by contrast, maintained ambipolar transport for days, which suggests that the multilayer character of VANS-grown silicene may be an advantage for the proposed hybrid.

### 3.6. Mechanistic basis of the three-way trade-off

Why can no single method satisfy all three criteria simultaneously? We argue that the answer is the intrinsic chemistry of silicon: silicon prefers sp^3^-like hybridisation, and the buckled honeycomb lattice of freestanding silicene is therefore metastable with respect to both 3D sp^3^ reconstruction and surface chemical attack. Every successful synthesis route must therefore impose an external stabilising influence. The Au(111) substrate is the most benign electronically of the available stabilizers, but its requirement for a noble-metal single crystal rules it out for industrial scale. Pristine freestanding monolayer silicene with the full Dirac cone signature will, for the foreseeable future, remain confined to the UHV laboratory.

## 4. Conclusions

A critical comparison of the four widely investigated silicene synthesis routes has been presented within a unified framework that imposes three application-driven criteria (scalability, ambient stability, and electronic fidelity) uniformly on every method. No single method simultaneously satisfies all three criteria, and silicene synthesis therefore remains an open research problem.

The electrochemical exfoliation route produces few-layer silicene-like nanosheets of ≈2.4 nm thickness and 30–100 nm lateral extent but is limited by kinetics and air instability.^22^ Epitaxial growth on Ag(111) delivers the highest crystallographic precision, yet it suppresses the Dirac cone^21,31^ and the controversy raised by Pan and Chou^28^ remains unresolved. The Au(111) substrate preserves the Dirac cone with reduced buckling,^30^ but molecular dynamics results require direct experimental confirmation. The PECVD route produces hydrogenated silicene with XPS-confirmed oxidation resistance.^24^ The Zintl phase route provides the strongest scalability, with VANS reducing reaction time from five days to minutes.^23^

We propose a hybrid synthesis strategy combining VANS growth with PECVD-derived Al_2_O_3_ encapsulation as a promising near-term pathway, and one of several stabilisation strategies currently under investigation. This is a testable hypothesis, with Tao *et al.*^45^ providing a direct cautionary lesson. Three priorities for future research are identified: (i) experimental validation of the Au(111) simulation results through direct STM, ARPES, and *in situ* Raman; (ii) direct experimental testing of the VANS + Al_2_O_3_ hybrid; and (iii) a dedicated experimental resolution of the silicene and Ag(111) structural controversy.

## Author contributions

Conceptualisation, M. M. U. and M. A. C.; investigation, M. M. U. and M. R. M.; writing – original draft preparation, M. M. U. and M. S. A. S.; writing – review and editing, M. A. C. and M. R. M.; supervision, M. A. C. All authors have read and agreed to the published version of the manuscript.

## Conflicts of interest

The authors declare that they have no known competing financial interests or personal relationships that could have appeared to influence the work reported in this paper.

## Data Availability

No primary research results, software or code have been included and no new data were generated or analysed as part of this review.
